# Gene-Gene Interaction and Functional Impact of Polymorphisms on Innate Immune Genes in Controlling *Plasmodium falciparum* Blood Infection Level

**DOI:** 10.1371/journal.pone.0046441

**Published:** 2012-10-12

**Authors:** Madhumita Basu, Tania Das, Alip Ghosh, Subhadipa Majumder, Ardhendu Kumar Maji, Sumana Datta Kanjilal, Indranil Mukhopadhyay, Susanta Roychowdhury, Soma Banerjee, Sanghamitra Sengupta

**Affiliations:** 1 Department of Biochemistry, University of Calcutta, Kolkata, West Bengal, India; 2 Cancer & Cell Biology Division, Indian Institute of Chemical Biology, Kolkata, West Bengal, India; 3 Centre for Liver Research, The Institute of Post-Graduate Medical Education & Research, Kolkata, West Bengal, India; 4 Department of Protozoology, The Calcutta School of Tropical Medicine, Kolkata, West Bengal, India; 5 Department of Pediatric Medicine, Calcutta National Medical College, Kolkata, West Bengal, India; 6 Human Genetics Unit, Indian Statistical Institute, Kolkata, West Bengal, India; London School of Hygiene and Tropical Medicine, United Kingdom

## Abstract

Genetic variations in toll-like receptors and cytokine genes of the innate immune pathways have been implicated in controlling parasite growth and the pathogenesis of *Plasmodium falciparum* mediated malaria. We previously published genetic association of *TLR4* non-synonymous and *TNF-α* promoter polymorphisms with *P.falciparum* blood infection level and here we extend the study considerably by (i) investigating genetic dependence of parasite-load on interleukin-12B polymorphisms, (ii) reconstructing gene-gene interactions among candidate *TLR*s and cytokine loci, (iii) exploring genetic and functional impact of epistatic models and (iv) providing mechanistic insights into functionality of disease-associated regulatory polymorphisms. Our data revealed that carriage of AA (P = 0.0001) and AC (P = 0.01) genotypes of *IL12B* 3′UTR polymorphism was associated with a significant increase of mean log-parasitemia relative to rare homozygous genotype CC. Presence of *IL12B+1188* polymorphism in five of six multifactor models reinforced its strong genetic impact on malaria phenotype. Elevation of genetic risk in two-component models compared to the corresponding single locus and reduction of *IL12B* (2.2 fold) and lymphotoxin-α (1.7 fold) expressions in patients'peripheral-blood-mononuclear-cells under *TLR4Thr399Ile* risk genotype background substantiated the role of Multifactor Dimensionality Reduction derived models. Marked reduction of promoter activity of *TNF-α* risk haplotype (C-C-G-G) compared to wild-type haplotype (T-C-G-G) with (84%) and without (78%) LPS stimulation and the loss of binding of transcription factors detected *in-silico* supported a causal role of *TNF-1031*. Significantly lower expression of *IL12B+1188* AA (5 fold) and AC (9 fold) genotypes compared to CC and under-representation (P = 0.0048) of allele A in transcripts of patients' PBMCs suggested an Allele-Expression-Imbalance. Allele (*A+1188C*) dependent differential stability (2 fold) of *IL12B*-transcripts upon actinomycin-D treatment and observed structural modulation (P = 0.013) of RNA-ensemble were the plausible explanations for AEI. In conclusion, our data provides functional support to the hypothesis that de-regulated receptor-cytokine axis of innate immune pathway influences blood infection level in *P. falciparum* malaria.

## Introduction

Infection with *Plasmodium falciparum* is still a major health problem worldwide, causing about 225 million new malaria cases each year [1]. Majority of the patients with parasite infection present febrile symptoms while a subgroup develops life-threatening complications such as severe malarial anemia (SMA) or cerebral malaria (CM). Malaria parasitemia which affects disease severity and transmission is controlled by a balance between depletion of red blood cells and immune clearance by T-helper cells, B lymphocytes, and cytokines as shown in murine model of *Plasmodium chabaudi* infection [2]–[4]. The role of innate immunity to restrict parasite growth in rodent model is further demonstrated by a recent study which shows when parasite dose saturates the capacity of innate response; experimentally enhanced innate immunity can control parasite density indirectly by depletion of RBCs [5]. In humans, hemoglobin degradation and heme detoxification by the obligate intracellular malaria parasite results in the formation of hemozoin (HZ) which along with malarial glycosylphosphatidylinositol (GPI) prime innate immune response by the production of pro-inflammatory cytokines through toll-like receptor mediated signaling [6], [7]. Two important functions of innate immunity in the defense against the parasite are (i) it triggers a battery of pro-inflammatory cytokines inhibiting rapid parasite growth and thereby limiting the onset of malaria pathology and (ii) it determines the type and efficiency of subsequent parasite specific adaptive immune responses through the cytokine mediators at later stages of infection [8]–[10]. An account of the etiological components of innate immunity and the mechanistic framework of their interactions will offer the much needed therapeutic alternatives for control of the global burden of malaria.

Genetic studies correlating malaria susceptibility with host immunity have a long history. Majority of the studies on the role of host genetics in determining malaria susceptibility have utilized population based case-control design and candidate or genome wide markers [11]–[20]. A general concern about the association study is that the alliance between disease phenotype and genetic loci remains tentative unless a functional causality is established. The major objective of this study is to understand the interactions and functional contribution of host genetic factors associated with a measurable phenotype, namely parasitemia, to extract an improved insight of host regulatory mechanisms controlling the blood infection level in *P. falciparum* mediated malaria. We have published a genetic association study of this phenotype analyzing fourteen single nucleotide polymorphisms (SNPs) located on genes encoding toll-like-receptor (*TLR*)-2, 4 and 9 and tumor necrosis factor-α (*TNF-α*) and lymphotoxin-α (*LTA*) and reported significant correlation of blood parasitemia with *TLR4* non-synonymous and *TNF-α* promoter polymorphisms in Indian patients with mild malaria [21]. Here we extend this analysis by investigating genetic association between polymorphic variability of *IL12B*, which encodes the *IL-12p40* subunit of *IL12*, with blood parasitemia. *IL12* is a heterodimeric pro-inflammatory cytokine with pleiotropic effects, acting as a potent immune-regulatory molecule and hematopoietic growth factor in infections caused by Plasmodium parasites [22]. It is produced by phagocytes and dendritic cells in response to pathogens through toll-like and other extracellular receptors. Physiologically the most important target cells of *IL12* are hematopoietic progenitors, NK cells and T cells for which it induces proliferation and production of type-1 cytokines (e.g. *IFN-γ*). *IL12* and *IFN-γ* together enhance activation and production of T_h_1 associated classes of immunoglobulin from B cells [23]. *IL12* is composed of a 35 kD subunit encoded by *IL12A* and 40 kD subunit encoded by *IL12B*. Polymorphisms in genes encoding both the subunits have been reported in a wide range of immune and inflammatory diseases including malaria [24]–[34].

Given the complexity of parasite biology and host immune system, it is unlikely that genetic variation of a single locus would provide an adequate explanation of inter-individual differences of host immune response which results in diverse clinical manifestations. To this end, identification of gene-gene interactions could enhance the power and accuracy of predicting disease outcome of a complex disorder. We have collated the genetic data on *IL12B* from the present report with that on toll-like receptors and cytokine loci from our previous study [21] to model the possible genetic interactions that may account for the differences in blood parasitemia in malaria patients. The functional relevance of receptor-cytokine epistatic models has been captured by analysis of receptor-genotype dependence of cytokine expression *in vivo*.

For a better description of genetic architecture of disease susceptibility and unambiguous identification of factors responsible for both causality and predisposition to a disease, functional appraisal of disease-associated polymorphisms is essential. There is widespread recognition that differences in gene expression may be an important source of phenotypic diversity in complex diseases [35]–[37] and that non-coding polymorphisms contribute to the variance and etiology of a trait by regulating the expression of nearby genes [38], [39]. To explore the plausible regulatory mechanisms exerted by cytokine SNPs we have characterized the allele specific events by studying their transcriptional differences in terms of reporter gene activities and allelic-expression-imbalance (AEI). Our study provides detailed insights into molecular effects of *cis*-regulatory variants in controlling cytokine gene expression in *P. falciparum* mediated malaria. However it underscores the possibility that this complex trait involves even more complex regulatory intricacies than previously anticipated.

## Materials and Methods

### Patient recruitment and Laboratory measures

A total of 293 mild malaria patients (age = 16–37 years) with *Plasmodium falciparum* infection were recruited from The Calcutta School of Tropical Medicine between September 2008 and January 2009 following WHO guidelines after obtaining the written informed consent from each study participant [40]. Patients with bacteremia, measles, acute lower respiratory tract infection, severe diarrhea with dehydration and other chronic or severe diseases such as cardiac, renal or hepatic diseases, HIV/AIDS were excluded from the study [21], [40]. Appropriate approvals have been obtained from Institutional ethical committees of University of Calcutta and The Calcutta School of Tropical Medicine, India. Parasitemia status of each patient was determined during their first visit to the clinic using Giemsa-stained blood smears and oil immersion microscopy. Detailed description of study samples and procedure for parasite enumeration can be found in our earlier report [21]. For genetic epidemiology, the patient pool from our previous study was used while for functional analyses case samples were enrolled (N = 64, age = 4–16 years) and registered in the year 2010 during July to September, from Calcutta National Medical College & Hospital, Kolkata, India as per WHO 2006 guidelines [40]. The blood samples for genetic and functional analyses were collected before any medical interventions.

### Genotyping IL12B polymorphisms

Genomic DNA was extracted from peripheral blood leukocytes using a QIAamp DNA Blood Kit (Qiagen, Hilden, Germany). *IL12B* promoter (rs17860508) and 3′UTR (rs3212227) polymorphisms were assayed through PCR followed by an allele specific restriction enzyme digestion. The primer pairs for genotyping were listed in Table S1. PCR amplification was carried out in 10 µl reaction mixtures containing 1 U AmpliTaq Gold™ DNA polymerase, 1.5 mM MgCl_2_, 250 µM of each dNTP, 5 pmoles of each primer (Sigma Aldrich, St. Louis, MO). The cycling parameters consisted of an initial denaturation at 96°C for 5 mins, followed by 40 cycles of denaturation at 96°C for 30 sec, annealing at 60°C for 30 sec, extension at 72°C for 30 sec, and then completed with a final extension at 72°C for 5 mins using a thermal cycler (Applied Biosystems® GeneAmp® PCR System 9700). PCR amplicons were digested with AluI (rs17860508) and TaqI (rs3212227) (Fermentas International Inc.) at 37°C and 65°C respectively according to manufacturer's protocol. Digestion patterns were analyzed by electrophoresis in agarose gel. The accuracy of the PCR-RFLP assay was confirmed for each locus by randomly selecting 10% of total samples and sequencing the PCR products on ABI Prism 3100 Genetic Analyzer using Big-Dye Terminator v3.1 (Applied Biosystems, Foster City, CA).

#### Statistical analysis

All the statistical tests were performed using SPSS version 10.0 and R-program (version 2.0 package). Associations between alleles/genotypes and parasite estimate were considered “strong” and “statistically significant” in cases where the P-value<0.05.

#### (a) Single Gene Analysis: Distribution of allele and genotype frequencies and genetic risk assessment of IL12B polymorphisms

The allele and genotype frequencies for *IL12B* SNPs were estimated by gene counting and Hardy–Weinberg equilibrium was evaluated by χ^2^ test using Haploview (http://www.broad.mit.edu/mpg/haploview/). Pairwise comparisons between log-transformed parasitemia and genotypic classes for *IL12Bpro* and *+1188* loci were analyzed by model based independent t test and ANOVA F-ratio. The results were further validated using non-parametric Wilcoxon Rank sum and Kruskal Wallis H tests. Bonferroni correction was done for multiple testing. For allele based risk assessment, the unprocessed parasitemia data was previously dichotomized into high (N = 88) and low (N = 205) parasitemic groups employing the two component mixture model and expectation-maximization algorithm and a threshold value of 7520 parasites/µl or 3.88 log-parasitemia was derived as the cutoff. Detailed procedure of this strategy was described earlier [21]. The proportion of the risk allele for each locus was compared between high and low parasitemic groups using a two-way contingency table.

#### (b) Multi-loci Analyses: Multifactor Dimensionality Reduction analysis

We adopted the multifactor dimensionality reduction (MDR) method upon merging the *IL12B* genotype data with those from our previous genetic epidemiologic study for identification of non-additive epistatic interactions [21], [41]–[46]. MDR is a non-parametric approach which converts multiple variables into a single attribute, thereby changing the representation space of the data. To run MDR, eight out of sixteen loci from the assembled dataset were selected as they showed an increasing/decreasing trend of log-parasitemia distribution across three genotypes which allowed us to code the genotypes into distinct risk groups. The “low”, “moderate” and “high” risk genotypic classes for each locus were designated as “0”, “1” and “2” while the “low” and “high” parasitemic groups were assigned as “0” and “1” respectively to depict the disease status. Since an equal number of sample size in high and low risk groups was a prerequisite for execution of MDR, the sample size of our analysis was kept to eighty eight, the number that represented in the high parasitemic group. Of the 205 individuals belonging to low parasitemic group, 100 distinct files each comprising of 88 individuals were generated using random numbers as seeds using R version 2.0 package to minimize the chance of detecting spurious results due to any bias. This was run on MDR software to obtain 100 independent outputs (version 2.0 beta 8.2). From eight genetic attributes (SNPs) namely *TLR4Thr399Ile* (rs4986791), *IL12B+1188* (rs3212227), *TNF-1031* (rs1799964), *TNF-857* (rs1799724), *TNF489* (rs1800610), *LTA80* (rs2239704), *LTA252* (rs909253), and *TLR9P545P* (rs352140), the possible multilocus genotypes were represented in a multidimensional contingency tables and classified as “high risk” if the ratio between two groups exceeded a threshold tested over the entire training data set. After reducing the dimensionality, all factor combinations were evaluated for their ability to classify the disease status in the training dataset and the best combination of factors with the minimum prediction error (1-testing balance accuracy) was calculated from the test dataset. Then the entire data was partitioned into 100 different subsets for cross-validation, from which ninety nine out of 100 subsets were assigned as a training balanced set (99/100) while the remaining one hundredth dataset (1/100) was termed as testing balanced set. To assign statistically meaningful gene-gene combinations, 100 MDR files were permutated 10,000 times using MDR permutation testing software (version 1.0 beta 2.0). The models with P value<0.05 and >95% average cross-validation consistency (CVC) were regarded as the best predictive models. To infer the genetic risk of the multifactorial models, we used odds ratio (OR) as a measure of association between genetic polymorphisms and blood infection intensity. The value of ORs was logarithmically transformed and standard errors were derived from the confidence intervals (CI). Genotype counts of the constituent loci between high and low parasitemic groups were used to calculate ORs and their corresponding 95% CIs for each gene-gene interaction model. A forest plot (blobbogram) was used to present the ORs and their 95% CIs estimated from the two-way interaction models where the odds ratio was denoted by a dot and the width of the horizontal line represented the 95% CI for the estimated OR.

### RNA extraction from PBMC fraction and cDNA synthesis

Peripheral blood mononuclear cells (PBMCs) were isolated from fresh whole blood collected from malaria patients using Histopaque 1077 (Sigma Aldrich, St. Louis, MO) double-gradient density centrifugation. Total RNA was extracted with TRI® Reagent (Sigma Aldrich, St. Louis, MO) and dissolved in DEPC treated water (Bioline). RNA (1 µg) samples were treated with 2 U of RNAse free DNAse I (Fermentas Life Sciences, UK) and incubated at 37°C for 30 mins to remove DNA contamination, reverse transcribed using random hexamers and High Capacity cDNA Reverse Transcription kit (Applied Biosystems Inc.). The optimized condition for cDNA preparation was 10 mins at 25°C, 120 mins at 37°C followed by heating at 85°C for 5 mins in a thermal cycler (Applied Biosystems® GeneAmp® PCR System 9700) and stored at −20°C.

### Quantitative Real Time PCR analyses

To scrutinize the genetic impact of *TLR4* non-synonymous polymorphism (rs4986791) on pro-inflammatory cytokine (*IL12B* and *LTA*) production, we compared the expression of *IL12B* and *LTA* genes under different *TLR4Thr399Ile* genotypic background. Since *TLR4Ile-Ile* and *TLR4 Thr-Ile* genotypes together and independently showed significantly low parasitemia compared to that of *TLR4Thr-Thr*, we pooled individuals having genotypes *Ile-Ile* with *Thr-Ile* genotypes and examined the pattern of cytokine gene expression with respect to patients harboring *TLR4Thr-Thr* genotype. The relative expression of *IL12B* was estimated for three +1188 genotypes by qRT-PCR using *18S rRNA* as an endogenous control. The sequences of oligonucleotides used in the expression analyses were listed in Table S1. A 1∶10 fold dilution of cDNA samples were used as the template and all qPCR reactions were carried out in a 10 µl reaction volume with 5 µl of SYBR Green Master Mix (Applied Biosystems) with optimized concentrations of specific primers using Applied Biosystems 7900HT Fast Real-Time PCR System. The thermal cycler was programmed for an initial denaturation step of 5 min at 95°C and followed by 40 thermal cycles of 30 sec at 95°C, 30 sec at 60°C and 30 sec at 72°C. The experiments were carried out in triplicate including the non template controls each time. Specificity of PCR amplification for each primer pair was confirmed by melting curve analysis [47] and the relative quantification (RQ) data was analyzed between groups using the ΔΔCt method. Statistical analysis on relative expression levels was estimated using Mann–Whitney U-test (with exact probabilities) for independent samples using SPSS (ver. 10.0., SPSS Inc. Chicago, IL).

### Allelic-Expression-Imbalance analysis

To examine the differential expression of *IL12B* 3′UTR A and C alleles, the peak heights of this transcribed SNP were compared in patients heterozygous (AC) for *IL12B+1188* polymorphism by resequencing both genomic DNA (gDNA) and complementary DNA (cDNA) counterpart. *IL12Bexp* primers (Table S1) were used to amplify and resequence the 167 bp *IL12B* region encompassing the polymorphic site. Sequencing was carried out on ABI Prism 3100 Genetic Analyzer using BigDye Terminator (BDT) v3.1 Cycle Sequencing Kit (Applied Biosystems, Foster City, CA) with 0.8 µl of the PCR product, and 5 pmoles of the primer in a 10 µl reaction mixture. The raw sequence files (.ABI) were analyzed by PeakPicker Software, originally developed and kindly provided by Dr. T. Pastinen's group [48]. Genomic DNA sequence from each sample was selected as reference and the corresponding cDNA was aligned with a default cutoff of 70%. The SNP was selected manually and the peak heights of the alleles were estimated using PeakPicker. The significant difference of *IL12B+1188* A/C allele ratio between the gDNA and cDNA was tested through a bivariate Sign test in SPSS version 10.0.

### Mapping of microRNA binding sites

To find out the potential microRNA binding sites within *IL12B* mRNA, the entire *IL12B* 3′UTR region was scanned using miRNA-target prediction databases such as TargetScan (www.targetscan.org/), miRBase (www.mirbase.org/), microRNA.org (www.microrna.org/), RegRNA (www.regrna.mbc.nctu.edu.tw/), MicroCosm (www.ebi.ac.uk/enright-srv/microcosm/). Target microRNAs were selected and compiled on the basis of its conserved seed match or a seed match with a higher context score. The thermodynamic stability of the mRNA-miRNA duplex was calculated using RNAHybrid (www.bibiserv.techfak.uni-bielefeld.de/rnahybrid/).

### Construction of reporter fusion plasmids

#### (a) For TNF-α promoter assay

Five *TNF-α* promoter haplotypes pertaining to four SNPs (*TNF-1031*, *TNF-857*, *TNF-308 and TNF-238*) were selected for functional evaluation. The 1.2 kb fragment was amplified using *TNFα_MluI_F.P* and *TNFα_XhoI_R.P* (Table S1) where MluI and XhoI recognition sequences were appended into the primers for efficient cloning into pGL3 vector. *TNF-α* haplotypes- T-C-G-G (Hap1), T-T-G-G (Hap2) and C-C-G-G (Hap3) were directly cloned into TA vector pTZ57R/T (InsTAclone™ PCR Cloning Kit, Fermentas) using the above primers by amplifying gDNA from patients harboring the homozygous genotypes for all four loci. Haplotypes: T-C-A-G (Hap4) and T-C-G-A (Hap5) were cloned by amplifying gDNA from patients harboring genotypes T/T-C/C-G/A-G/G and T/T-C/C-G/G-G/A respectively, followed by screening the clones with restriction endonucleases NcoI for *TNF-308* locus and BamHI (NEB Inc., Bethesda) for *TNF-238* respectively. DNA amplification protocol was conditioned with an initial denaturation at 95°C for 5 min, followed by 35 cycles of denaturation at 95°C for 1 min, annealing at 60°C for 1 min, extension at 72°C for 1 min and a final extension of 15 min at 72°C in a thermal cycler (Applied Biosystems® GeneAmp® PCR System 9700). The unique haplotypes from the TA constructs were subsequently double-digested, purified using QIAquick Gel Extraction Kit (Qiagen, Hilden, Germany) and subcloned in the upstream of pGL3-Basic vector (kindly gifted by Dr. Susanta Roychowdhury). The integrity of each polymorphic locus and directionality of all the resulting constructs were confirmed by sequencing using both vector and insert specific internal primers. Next, the reporter gene expressions were measured in three different cell lines under the presence and absence of endotoxin lipopolysaccharide (LPS) stimulation using Luciferase Reporter Assay System (Promega, Madison, WI).

#### (b) For IL12B3′UTR assay

*IL12B* 3′UTR sequence (1047 bp) encompassing the A+1188C polymorphic site was amplified using gene specific primers (Table S1). XhoI and NotI (Fermentas International Inc.) restriction endonuclease recognition sites were incorporated in the oligos for directional cloning. Allele specific *IL12B* 3′UTR containing the target sites for the microRNAs was cloned into pTZ57R/T vector (InsTAclone™ PCR Cloning Kit, Fermentas) followed by subcloning into pSiCHECK2 dual luciferase reporter plasmid (kindly gifted by Dr. Soma Banerjee) in frame to the 3′ end of the cDNA encoding *Renilla reniformis* luciferase to produce pSiCHECK2-IL12B+1188A and pSiCHECK2-IL12+1188C constructs. Approximately 100 bp upstream and downstream sequences flanking the 70 nucleotide pre-miR sequences (www.genecards.org/) were amplified for hsa-miR-545, hsa-miR-1284, hsa-miR-23a and hsa-miR-23b using appropriate primers (Table S1) and cloned into pRNAU6.1 vector (kindly gifted by Dr. Soma Banerjee). The amplicons were inserted into pRNAU6.1 vector by double digestion with BamHI and HindIII (New England Biolabs, UK Ltd.). The orientation of the recombinant vectors was checked by sequencing. Allele specific transcript stability and miRNA-mediated *IL12B* 3′UTR expression were examined with SYBR Green based qRT-PCR analysis and by Dual-Luciferase Reporter (DLR) Assay System (Promega, Madison, WI) respectively.

### Cell culture, Transient transfection, Luciferase and mRNA stability assay

HepG2 (hepatocellular adenocarcinoma), HEK293 (human embryonic kidney), HCT116 (colon carcinoma) and U937 (human leukemic monocyte lymphoma) cells were obtained from National Centre for Cell Science, Pune, India and maintained in DMEM medium containing 10% (v/v) fetal calf serum (Gibco BRL, Life Technologies, Grand Island, USA), 100 units/ml penicillin, 100 mg/ml streptomycin in a humidified 5% CO_2_ atmosphere. U937 cells were cultured in RPMI 1640 supplemented with 5 mM glutamine and 10% (v/v) heat-inactivated fetal calf serum (Gibco BRL, Life Technologies, Grand Island, USA) at 37°C in 5% CO_2_ atmosphere. Cells were seeded 14–16 hrs before transfection.

#### (a) TNF-α promoter haplotype assay

HepG2 cells (1.0×10^5^) were plated in 12-well plates with DMEM/10% FCS medium and were transiently transfected with 0.2 µg/ml of each of the *TNF-α* promoter constructs (Hap1–Hap5) or empty pGL3-Basic vector cotransfected with 0.5 µg/ml pSV-β-galactosidase vector (Promega, Madison,WI) using Lipofectamine 2000 Reagent (Invitrogen, Life Technologies, Carlsbad, USA) according to the manufacturer's protocol. pGL3-Basic vector (Promega, Madison, USA) was used as control for luciferase assay and β-galactosidase vector (Promega, Madison, USA) was used as transfection control. To determine the optimum concentration of LPS (Sigma Aldrich, St. Louis, MO) for stimulation assay, a dose response experiment was performed with cells transiently transfected with *TNF*-Hap1 (T-C-G-G) followed by a stimulation of LPS solutions with five different concentrations such as 250 ng/ml, 500 ng/ml, 1 µg/ml, 1.5 µg/ml to 2 µg/ml and luciferase activities were measured. 0.2 µg of each of the five reporter plasmids (Hap1–Hap5) were transiently transfected. Thirty six hours post transfection, cells were stimulated with optimized dose of LPS for 4–6 hr and cellular extracts were prepared according to the manufacturer's instructions. Luminescence was measured as relative light units (RLU) in GloMax 20/20 Luminometer (Promega, Madison, USA) using 15 µl of cell supernatant. To normalize the luciferase activity, total protein concentration in each lysate was measured by the standard Bradford (BioRad) method. Transfection normalization was performed by β-galactosidase assay colorimetrically at 420 nm using O-nitrophenyl-β-D-galactopyranoside (ONPG) as substrate (Promega, Madison, WI) in JASCO V-630 spectrophotometer. Measurements of mean ± S.D were taken in triplicate. Fold change represents the difference in promoter activity for variant *TNF-α* haplotypes in comparison to the wild-type promoter (Hap1). All the assays were done in triplicates and repeated at least for three times. Similar experiments were also performed in HEK293 and HCT116 cell lines both under LPS stimulated and unstimulated conditions.

#### (b) IL12B mRNA stability assay by actinomycin-D

Actinomycin-D (2-amino-N,N′-bis[(6S,9R,10S,13R,18aS)-6,13-diisopropyl-2,5,9-trimethyl- 1,4,7,11,14-pentaoxohexadecahydro-1H-pyrrolo[2,1-i] [1], [4], [7], [10], [13] oxatetraazacyclohexadecin- 10-yl]-4,6-dimethyl-3-oxo-3H-phenoxazine-1,9-dicarboxamide), a polypeptide containing antibiotic, can inhibit transcription by binding tightly and specifically to double-helical DNA [49], [50]. It has been extensively used as a highly specific inhibitor for the formation of new *de novo* mRNA synthesis in both prokaryotic and eukaryotic cells [51], [52]. In this experiment, HepG2 cells were transiently transfected with plasmid DNA containing the *IL12B* 3′UTR fused to pSiCHECK2 vector, as described above. To determine the optimal concentration of actinomycin-D (SRL, Mumbai, India) in our experimental system, a range of concentrations of actinomycin-D (0.1–10 µg/ml) were studied. Twenty-four hours post-transfection with wild-type +1188AUTR construct, actinomycin-D was added into the media and the cells were harvested at different time points, i.e., 0, 1, 2, 4, 6, 18 and 24 hrs. Total RNA was extracted from these cells using TRIzol® Reagent (Invitrogen Life Technologies) and *IL12B* mRNA level was determined by RT-PCR and normalized with *GAPDH* and *18S rRNA* genes. RNA isolated when actinomycin-D was just added was denoted as the 0-hr time point and served as negative control. It was compared with samples obtained at different time points following actinomycin-D addition to select the adequate incubation time where maximum change in expression could be found for both alleles. To compare relative mRNA stability of *IL12B* 3′UTR A and C containing constructs, HepG2 cells were transfected and *de novo* transcription was inhibited with the optimal concentration of actinomycin-D. Cells were collected after 24 hrs of actinomycin-D treatment and the mRNA levels of the transfected gene (IL12B-3′UTR/Luc) were determined by quantitative real-time PCR.

#### (c) IL12B 3′UTR-microRNA interaction assay

HepG2 cells (1×10^5^) were transiently transfected with 0.1 µg/ml of the *IL12B+1188* wild type (A) and variant allele (C) containing pSiCHECK2 dual reporter constructs alone and cotransfected with either 0.2 µg/ml of empty miR-vector (pRNAU6.1) and/or with the pre-miRNA (hsa-miR-545, hsa-miR-1284, hsa-miR-23a and hsa-miR-23b) cloned in pRNAU6.1. Forty eight hours later, cells were lysed; Firefly and Renilla luciferase activities were evaluated using dual luciferase reporter assay system (Promega, Madison, WI) in a GloMax 20/20 Luminometer. Renilla luciferase activity was normalized with respect to Firefly luciferase activity and total protein produced was estimated by Bradford method. All the transfection assays were done in triplicate and repeated thrice. Measurements of mean ± S.D were taken in triplicate. The pairwise comparisons between each mRNA-miRNA interactions were tested using Student-t test in GraphPad Prism (http://www.graphpad.com).

### Bioinformatic analysis: SNP based DNA and RNA structural rearrangements

#### (a) TNF-1031 polymorphism associated DNA two dimensional configurations

DNA folding was performed with MFOLD version 3.0 (www.bioinfo.math.rpi.edu/~mfold/dna/) using the DNA energy rules in structure-melting simulations, with default parameters [53], [54], i.e., having 1 M Na+ concentration (and 0.0 M Mg^2+^) at 37°C. Outputs were generated in the form of structure plots based on minimum free energy (MFE) calculations, single strand (ss) frequency count and energy dot plots [55]. In addition, to identify the transcription factor binding sites (TFBS) in the genomic sequence; TFBind (http://tfbind.hgc.jp/) [56] was used to scan the 101 bp long sequence flanking the promoter SNP (rs1799964) located at 51^st^ position. This positional pattern detection tool is able to attain high sensitivity and specificity of detection by capturing the dependencies between nucleotide positions within the TFBS.

#### (b) IL12B+1188 derived RNA structural ensemble prediction

In an attempt to improve our understanding of the structure and function relationship of the *IL12B* 3′UTR polymorphism on RNA turnover and stability, we performed an *in silico* structural analysis including the wild type and variant form of the locus on *IL12B* mRNA. An mRNA is unlikely to adopt a single and stable conformation, but it exists in a population of structures [57]; therefore instead of evaluating the minimum free energy (MFE) values of biological sequences, those of randomized sequences are now taken into account. As RNA secondary structure might affect the functionality of component regulatory elements, RNAfold program (http://rna.tbi.univie.ac.at/cgi-bin/RNAfold.cgi) was employed to predict secondary structures under default parameters and the folding temperature was fixed at 37°C [55], [58], [59]. In addition to the minimum free energy approach using RNA MFOLD and RNAfold, we adopted a partition function calculation method which evaluates the entire ensemble of possible RNA conformations for a given sequence and computes the effect of single nucleotide polymorphisms on RNA structural ensemble using SNPfold (http://ribosnitch.bio.unc.edu/snpfold/SNPfold.html). The structures were also generated with Sfold (http://sfold.wadsworth.org/) algorithm that computes equilibrium partition functions for all substrings of an RNA sequence based on the Turner thermodynamic parameters [60], [61]. Base-pair distances were used [62], [63] as the basis for evaluating dissimilarity between structures where the “centroid” in the entire structure ensemble space has the shortest total base-pair distance [62].

## Results

The patients participating in this genetic association study were recruited according to WHO 2006 guidelines [40]. All of them displayed mild febrile symptoms, the summary of demographic, clinical and laboratory characteristics of the study participants were presented earlier [21]. In short, the crude parasitemia data derived from thick and thin blood smears deviated from normal behavior (skewness = 3.671), it was processed with log-transformation (skewness = 0.433) and regressed with respect to the covariate, age (P value of correlation between age and parasite load was 0.074). For allele based genetic analyses, cases were stratified into high and low parasitemic groups on the basis of a statistically derived threshold value of 7520 or 3.88 in terms of crude and log-parasite counts per µl respectively. The above threshold value was obtained employing a two component mixture model and Expectation Maximization algorithm [21]. No significant differences were observed in the distribution of age (P = 0.2632), gender (P = 0.3728), Hemoglobin count (P = 0.2971) and in average body weight (P = 0.3901) between the high and low parasitemic groups. Only peripheral parasitemia was found to be significantly different (high = 15227±11014; low = 2715±1705, P<0.0001) between the groups.

### Genetic analysis with IL12B polymorphisms

We studied the genetic variants located in the promoter and 3′UTR region of the gene encoding *IL12B* by PCR based RFLP method. Two biallelic markers; rs17860508 (designated as *IL12Bpro*): a complex promoter insertion/deletion polymorphism -/G/GC/TTAGA/TTAGAG situated at −2703 bp upstream of the transcription initiation site and rs3212227 (designated as *IL12B+1188*): an A to C substitution in the 3′UTR region at *+*1188 position of *IL12B* gene were selected to examine their probable association with the blood infection intensity in two hundred and ninety three patients exhibiting *Plasmodium falciparum* mild malaria from Eastern India.

### Genetic association between IL12B SNPs and peripheral parasite load: Genotype & allelic

**Figure 1:**
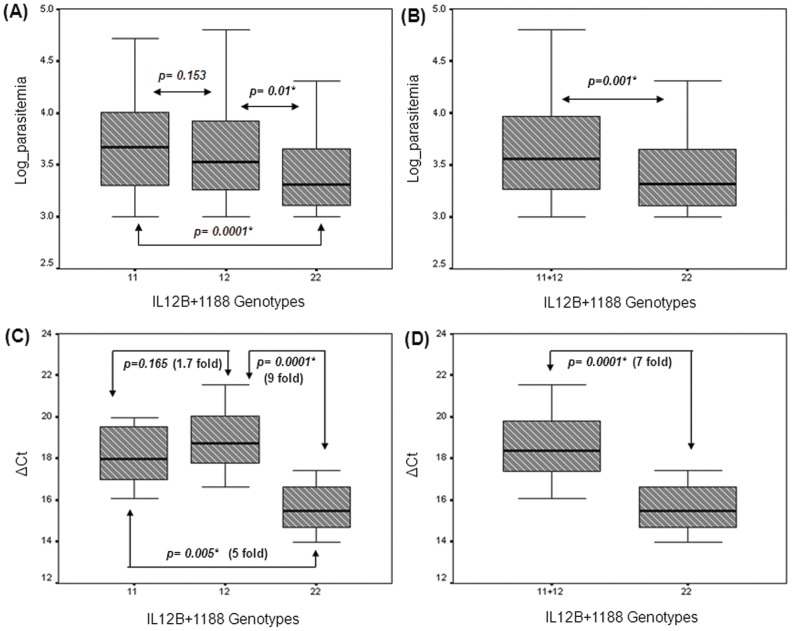
Association between *IL12B+1188* genotypes with blood infection level and *IL12B* gene-expression represented in Box plots. (A) Diagram represented the distribution of log-parasitemia across three genotypes: 11 (AA), 12 (AC) & 22 (CC) and (B) Diagram represented the comparison of log-parasitemia of minor homozygous genotype (CC) with AA and AC genotypic groups pooled. Statistical significance between pairwise comparisons was mentioned. (C) The ΔC_t_ distribution of *IL12B* gene expression across AA (N = 24), AC (N = 28) and CC (N = 12) genotypes and (D) comparison of gene expression between *IL12B+1188CC* genotype and with that of AA and AC individuals pooled together determined by quantitative real time PCR. Statistical significance was determined by the Mann Whitney U test. P values and fold changes obtained for each pairwise combination were appended in each plot. (*) indicates these differences to be statistically significant. The bottom, middle line, and top of each box correspond to the 25^th^ percentile, median, and the 75^th^ percentile, respectively. Bars extend to the lowest value and to the highest value of each group.

*IL12Bpro* (MAF: 0.357±0.002) and *IL12B+1188* (MAF: 0.404±0.002) were found to be highly polymorphic in the study population and there was no deviation from Hardy-Weinberg equilibrium (P values = 0.3934 and 0.6799 for *IL12Bpro* and *IL12B+1188* respectively). The distribution of genotypes for *IL12Bpro* and *IL12B+1188* loci were summarized in Table 1. For model dependent association analysis, the crude parasitemia data collected from malaria patients was log transformed and subjected to linear regression with respect to age in order to obtain the standardized residuals which were then used to examine the influence of genotypes at each locus on the parasite load under different genetic models. To find out the genetic risk conferred by a SNP, *IL12Bpro* and *IL12B+1188* were independently evaluated as covariate using three genetic models viz., dominant ((11+12) vs 22), recessive (11 vs (12+22)), and additive (11 vs 12 vs 22), where 1 represents as the risk allele using independent t-test and ANOVA (SPSS v10.0). The results of parametric analyses were substantiated by Wilcoxon Rank Sum and Kruskal Wallis H tests for two and three way comparisons respectively using the crude parasitemia data. Box-plot diagrams (Figure 1A & B) displayed a gradual decline in parasitemia across three genotypes of *IL12B+1188* locus in which AA (11), AC (12) and CC (22) had the highest, intermediate and lowest median values respectively. Significant differences were obtained for the comparison 11 vs 12 vs 22 using ANOVA test (P value = 0.001) and for the following pairwise genotype comparisons namely (11+12) vs 22 (P value = 0.001); 12 vs 22 (P value = 0.014) and 11 vs 22 (P value = 0.0001) using independent t-test presented in Table 1. The P values in the parentheses were obtained after Bonferroni correction. Non-parametric statistical tests yielded similar trends in the results (Table 1). The mean log parasitemia of 11 (AA = 3.68±0.45) genotype and the heterozygote genotype (AC = 3.58±0.41), separately and jointly, differed significantly (P value = 0.001) from that of 22 (CC = 3.40±0.35), while the difference between mean parasite loads representing genotypes 11 and 12 was not statistically significant (P value = 0.153) suggesting a dominant effect of 1 (A) allele over allele 2 (C) for the *IL12B+1188* locus to be the most likely explanation. Similar statistical tests were conducted on IL12B*pro* (rs17860508), however none of the comparisons were significant for this polymorphism. The linkage disequilibrium estimate (r^2^) between these two loci that were 17,249 bp apart was 0.06 (P = 0.3052) suggesting an independent segregation of the promoter and UTR polymorphisms in our population.

**Table 1:** Genetic association of *IL12B* promoter and 3′UTR polymorphisms with parasitemia at genotypic level.

Locus ID (MAF^a^± SD)	Genotype	Cases (N = 293) (Mean log parasite ± SD)	Pairwise Combination	ANOVA F-ratio	t-test (P-value)	K-W Test χ^2^ (P-value)	Wilcoxon Rank Sum test (P-value)
*IL12Bpro* (0.357±.002)	11	125 (3.64±0.43)	11 vs 12	1.761 (0.174)	0.118	3.430 (0.180)	0.091
	12	127 (3.55±0.44)	12 vs 22		0.672		0.965
	22	41 (3.52±0.35)	11 vs 22		0.113		0.189
	11+12	252 (3.59±0.43)	11+12 vs 22		0.298		0.298
	12+22	168 (3.53±0.39)	11 vs 12+22		0.062		0.062
*IL12B+1188* (0.404±.002)	11	106 (3.68±0.45)	11 vs 12	7.732 (0.001*)	0.153	13.164 (0.001*)	0.097
	12	137 (3.58±0.41)	12 vs 22		0.014*		0.01*
	22	50 (3.40±0.35)	11 vs 22		0.0001*		0.0001*
	11+12	243 (3.62±0.43)	11+12 vs 22		0.001*		0.001*
	12+22	187 (3.53±0.45)	11 vs 12+22		0.01*		0.01*

a *MAF denotes minor allele frequency and SD denotes standard deviation.*

* *P-Value<0.05.*

The allele based risk assessment for *IL12B+1188* was performed by stratifying the patients into “low” (N = 205) and “high” (N = 88) parasitemic groups. A detail of this procedure was described elsewhere [21]. The proportions of two alleles A and C of *IL12B+1188* compared using a two way contingency table were significantly different between the groups (P<0.001). The odds ratio (OR) of comparison (1.887, 95% CI = 1.3–2.78) was due to an excess of allele A in the “high” parasitemic group (Table 2). Taken together, the results of the genetic association study supported a strong influence of *IL12B+1188* polymorphism on *P. falciparum* infection load in our samples at both genotype and allelic levels.

**Table 2:** Allele based risk assessment in patients with high and low parasite load.

Locus ID	Allele	High Parasitemic group (N = 88) Frequency	Low Parasitemic group (N = 205) Frequency	OR^b^ (95% cCI)	χ^2^ (P-value)
*IL12Bpro*	1	0.68	0.63	1.28(0.88–1.85)	1.39 (0.238)
*IL12B+1188*	**1**	0.70	0.55	1.89(1.30–2.78)	(0.001*)

*Risk allele was boldfaced. N = total number of sample. χ2-test was used to estimate the differences between the allele frequencies.*

b *OR = odds ratio.*

c *CI = confidence interval.*

* *P-Value<0.05.*

### Evaluation of gene-gene interactions: Multilocus analyses and MDR

**Figure 2:**
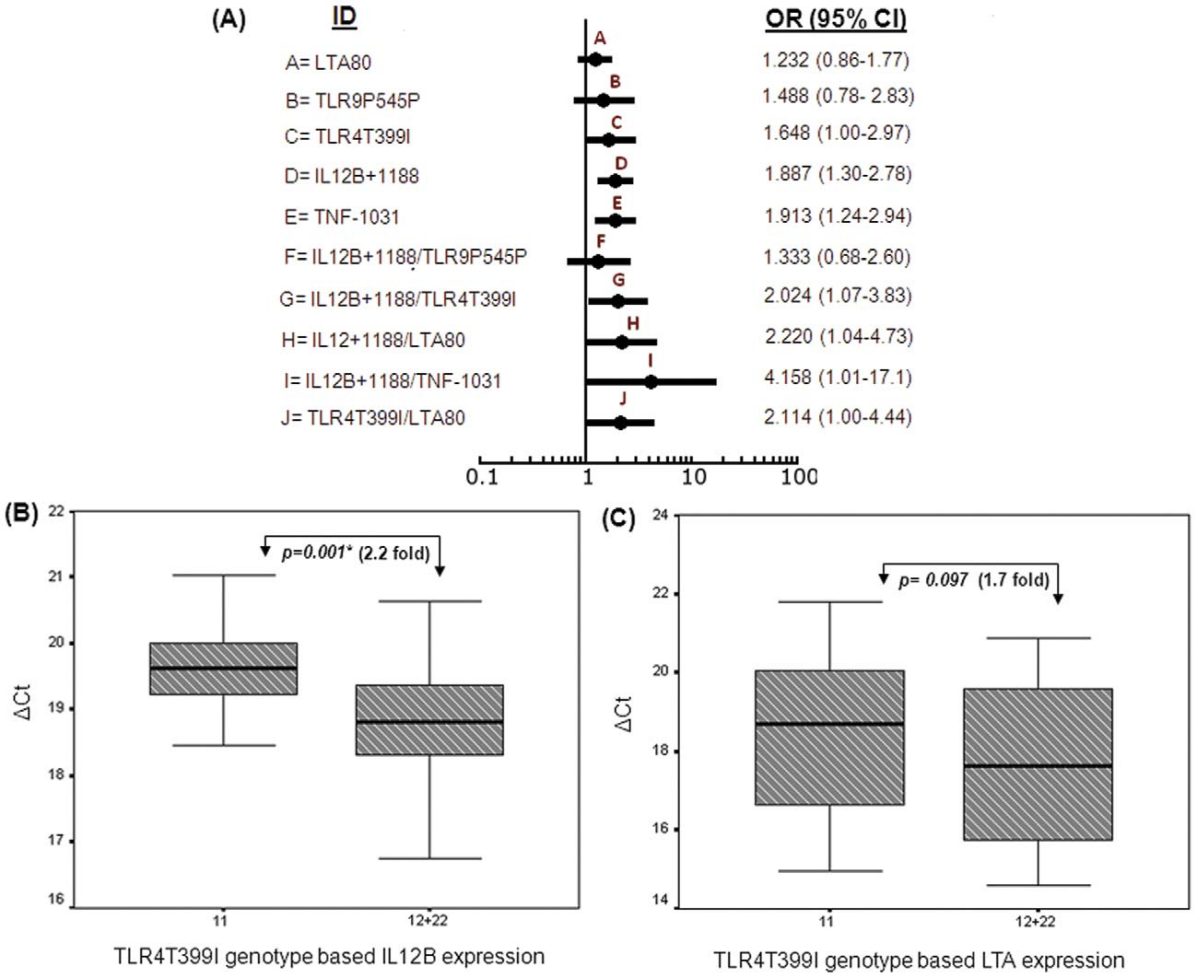
Genetic and functional association of multifactor models obtained by Multifactorial Dimensionality Reduction analysis. (A) Forest plots presented the comparison of risk estimates in terms of odds ratio (OR) and 95% confidence interval (CI) for significant gene-gene interaction models and component single loci. The risk corresponding to each single and two factor models was denoted by a dot and the horizontal lines represented odds ratio and 95% CI respectively. The model IDs (A–J) and respective ORs (95% CI) were given at the left and right side of each dot in the forest plot. (B) *IL12B* and (C) *LTA* gene expression in patients' PBMCs classified according to *TLR4Thr399Ile* genotype status by real time quantitative PCR. Distribution of ΔC_t_ was plotted and compared between the genotypic groups. Statistical significance was determined by Mann Whitney U test. P values and corresponding fold changes obtained for each pairwise comparison were shown in the box plots. (*) indicates these differences to be statistically significant.

The genetic etiology of complex diseases is considered to involve interactions among multiple genetic variants and environmental conditions. Multifactorial Dimensionality Reduction [41] was performed to explore potential gene-gene interactions by combining the *IL12B* genotype data of this study with our previous one [21]. We identified five two-factors (Model I–V) and one three-factor (Model VI) gene-gene interaction models with an average cross-validation consistency (CVC) >95% and a P value<0.05 using MDR permutation testing program. The gene-gene partners of MDR models were summarized in Table 3. The interacting models that satisfied the above criteria, included *TLR4Thr399Ile* (rs4986391) and/or *IL12B+1188* (rs3212227) in combination with either *TNF-1031* (rs1799964) or *LTA80* (rs2239704) or *TLR9P545P* (rs352140). The average prediction errors (PE) for these models ranged from 0.305 to 0.366. To estimate the genetic risk under a two-factor model, the proportions of individuals harboring risk genotypes for partner loci, were compared between the low and high parasitemic groups. Since *IL12B+1188* was present in five out of six genetic models the odds ratio of the second locus was estimated under *IL12B+1188* risk (AA+AC) background. The comparison of odds ratio (OR) and 95% confidence intervals (CI) of two-way and the constituent interacting partners were shown in the Forest plot (Figure 2A). The proportion of risk genotypes of three SNPs, namely *TLR4Thr399Ile* (Model I: OR = 2.024, 95% CI 1.07–3.83, P value = 0.028), *TNF-1031* (Model III: OR = 4.158, 95% CI 1.01–17.1, P value = 0.033) and *LTA80* (Model V: OR = 2.22, 95% CI 1.04–4.73, P value = 0.034) in association with *IL12B+1188* elevated remarkably in high parasitemic groups compared to the single SNP analyses (Figure 2A; ID: G, I and H). For Model II, odds ratio for *LTA80* (OR = 2.113, 95% CI 1.0–4.44, P value = 0.047) under *TLR4* risk background (Thr-Thr) was also increased (Figure 2A; ID: J). Notably, the difference in mean parasitemia for *LTA80* between the genotype comparison (11+12) vs 22 was not significant (OR = 1.232, 95% CI 0.861–1.766, P value = 0.074) when the locus was analyzed alone (Figure 2A; ID: A). In short, except for *TLR9P545P*, all two-way epistatic models yielded higher risk of developing high parasitemia compared to the single marker analyses. Model VI: *TLR4Thr399Ile/IL12B+1188/LTA80* did not modify the risk significantly due to reduction of sample size in the high parasite group when three loci were pooled.

**Table 3:** Best predictive gene-gene interaction models identified by multifactor dimensionality reduction analysis.

Model No.	Best Predictive Interaction Model^a^	Prediction Error (1-TBA^b^)	10,000 Permutations P-value	c CVC (in %)
I	*TLR4Thr399Ile/IL12B+1188*	0.318	0.024*	95
II	*TLR4Thr399Ile/LTA80*	0.358	0.033*	100
III	*IL12B+1188/TNF-1031*	0.341	0.013*	100
IV	*IL12B+1188/TLR9P545P*	0.329	0.005*	100
V	*IL12B+1188/LTA80*	0.366	0.042*	100
VI	*TLR4Thr399Ile/IL12B+1188/LTA80*	0.305	0.031*	100

a *The best model was selected as the one with the minimum prediction error and maximum CVC.*

b *TBA corresponds to the testing balanced accuracy defined as the prediction error (PE) = 1-TBA.*

c *CVC corresponds to cross validation consistency.*

* *P values for gene-gene interaction models were calculated after 10,000 permutations in MDRpt software.*

*TLR4* encodes an extra-cellular receptor present in the antigen presenting cells (APCs) and perceives the malaria antigens to trigger a complex cascade of downstream signaling events which ultimately culminates into the production of several pro-inflammatory cytokines including *IL12* and *LTA* [64], [65]. To translate functional impact of the above MDR derived gene-gene interaction models (Model I and II) in terms of *TLR4* mediated signaling efficacy, we compared the expression levels of *IL12B* and *LTA* genes under *TLR4Thr399Ile* risk (11; N = 16) and non-risk (12+22; N = 33) genotype backgrounds using quantitative RT-PCR analyses. Patients harboring heterozygous and the rare homozygous genotypes (Thr-Ile and Ile-Ile) showed significantly increased (2.2 fold, Mann Whitney P value = 0.001) expression of *IL12B* gene (^median^ ΔC_t_±S.D = 18.80±0.87) compared to those with homozygous (Thr-Thr) genotype (^median^ ΔC_t_±S.D = 19.63±0.74) as shown in Figure 2B. The difference in *LTA* expression between *TLR4Thr399Ile* risk and non-risk genotypic backgrounds did not cross the threshold of statistical significance by non-parametric Mann Whitney U test (1.7 fold, P value = 0.097), though the expression of *LTA* gene (Figure 2C) was higher in *TLR4*Thr-Ile and Ile-Ile genotype background (^median^ ΔC_t_±S.D = 17.66±1.7) compared to that estimated in *TLR4*Thr-Thr risk environment (^median^ ΔC_t_±S.D = 18.43±1.5). Taken together, our results provided the genetic (Figure 2A) and functional (Figure 2B & C) validations of MDR gene-gene interaction models.

### Functional analysis: TNF-α promoter and IL12B 3′UTR polymorphisms

In addition to provide functional support to the statistically inferred epistatic models, we attempted to delineate the molecular role of *TNF-α* promoter and *IL12B* 3′UTR polymorphisms using a combination of methods including quantitative RT-PCR, allelic-expression-imbalance, mRNA stability assay, luciferase based promoter (*TNF-1031*) and 3′UTR (*IL12B+1188*) activity analyses, bioinformatic prediction of putative mRNA-miRNA binding interactions and SNP based DNA, RNA secondary structure assessment.

#### (a) Efficiency of TNF-α promoter haplotypes under LPS stimulated and unstimulated conditions: Reporter gene assay and Bioinformatic support

**Figure 3:**
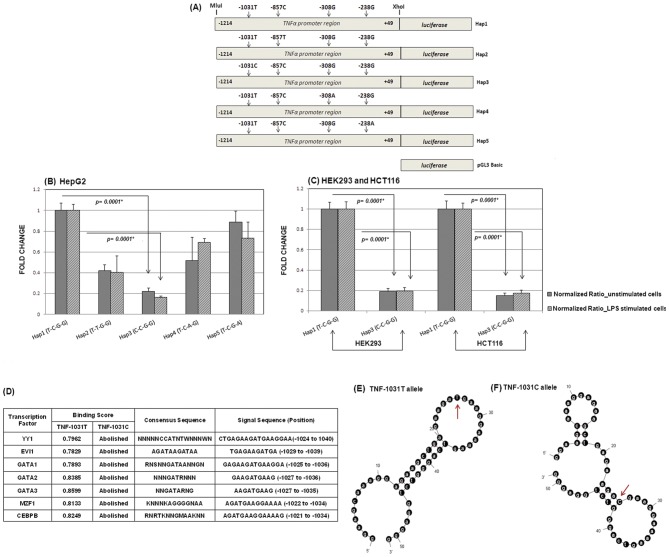
Results of *TNF-α* promoter assay. (A) Schematic representation of reporter gene constructs for five *TNF-α* promoter haplotypes (Hap1–Hap5) used for transfection assays. (B) HepG2 cells (1×10^5^ cells/ml) were transiently transfected with all promoter constructs and relative luciferase activities in supernatants were measured alone (light grey) or with cells stimulated with 250 ng/ml of LPS (dark grey) after 48 hours. Activity of wild-type promoter haplotype TNF-α-Hap1 served as reference and set at one, and the variant constructs were expressed as fold changes in relation to this. Statistical significance for all pairwise comparisons was done by t-test. P values of significant differences between haplotype expressions were marked with asterisks (*). (C) Similar experiments were performed with wild-type Hap1 and variant Hap3 constructs in HEK293 and HCT116 cells and corresponding differences in promoter activities were measured. (*) indicates the significant comparisons given as P values. (D) Putative transcription factor binding profiles for *TNF-1031* T allele summarizing the TFs with their binding scores, consensus and signal sequences which were completely abolished in presence of the variant (C) allele. (E & F) MFOLD derived representative DNA secondary structures encompassing 50 bp *TNF-α* promoter sequence. The numbers indicate the base position while the arrowhead marks the −1031 site.

To explore whether *TNF-1031* polymorphism influenced the basal rate of transcription of the *TNF-α* gene, haplotypes pertaining to a 1.2 kb fragment spanning the proximal promoter SNPs at positions −1031, −857, −308 and −238 were subjected to reporter gene based assay. Notably, haplotypes were derived using Haploview and out of the 16 possible haplotypes, only six were observed in our population with haplotype T-C-G-G (1-1-1-1) showing the highest frequency in both high and low parasitemic groups. This reference haplotype as well as four other haplotypes that differed from the former at a single nucleotide position were cloned. The frequency of the variant haplotypes were >3% in the patient samples. Figure 3A showed the map of the promoter haplotypes cloned and assayed for reporter gene activity in HepG2 cells both in presence and absence of LPS, which was known to be one of the parasite-associated-molecular-patterns (PAMPs) recognized by *TLR* molecules [66]. Fold changes in variant promoter (Hap2-5) activities with respect to reference haplotype Hap1 were indicated in Figure 3B. All four variant haplotypes (Hap2-5) showed decrease in luciferase activity both in presence (250 ng/ml) of LPS (27–84%) and in absence (12–78%) of the antigen (Figure 3B). The maximum reduction in luciferase activity [78% (−LPS) and 84% (+LPS), P<0.001] was observed for Hap3 construct (C-C-G-G) which harbored the risk allele C for *TNF-1031*, the locus that showed significant genotypic and allelic association with blood parasite infection [21]. Our comparative promoter assay identified Hap1 and Hap3 as the strongest and weakest promoter among the five different *TNF-α* haplotypes in HepG2 cell line in three independent experiments. Similar experiments were performed in HEK293 and HCT116 in triplicate to compare the promoter activities of Hap1 and Hap3 (Figure 3C). A consistent reduction of the reporter gene expression under stimulated and unstimulated conditions (80%) for Hap3 was noted in both the cell lines. The control pGL3-Basic vector showed very low levels of relative luciferase activity in both the unstimulated and LPS-stimulated cells.

Scanning of a 101 bp region flanking 50 bp in the either direction of *TNF-1031* locus by TFBind program revealed that T to C transition caused loss of binding of several transcription factors. A list of the transcription factors that putatively showed higher binding affinity for the T allele given by binding scores, corresponding consensus and signal sequences with relative nucleotide positions upstream of *TNF-α* coding sequence were presented in Figure 3D. The possibility of altered DNA-protein interactions in terms of changes in local secondary structure due to the sequence variation at −1031 was examined using MFOLD web server (Figure 3E and F). Out of the seventeen possible computed foldings, thirteen (76.5%) with T at −1031 position were found to be base paired while twenty (83.3%) out of twenty four possible conformations had variant C allele locked in stems. The minimum free energy (MFE) conformations constructed using 50 bp local DNA sequence encompassing *TNF-1031* were shown for wild type and variant alleles (Figure 3E & F). Results of these *in-silico* comparisons indicated a relative reduction of accessibility of a transcription factor when the risk allele was present in the sequence. Taken together, the polymorphism at −1031 of *TNF-α* caused a loss of promoter activity as well as transcription factor binding as per our reporter gene assay and bioinformatics analyses respectively.

#### (b) Impact of IL12B+1188 (rs3212227) genotypes and alleles on gene expression: a quantitative evaluation

*IL12B+1188* polymorphism showed strong genetic association with the blood parasite level in the malaria patients both at genotype and allele levels. To test the hypothesis that this 3′UTR polymorphism may modulate *IL12B* transcript turnover, relative expression of *IL12B*-mRNA was quantified using the cDNA synthesized from mRNA extracted from peripheral blood mononuclear cell (PBMC) fraction of whole blood collected from individuals harboring three different genotypes namely AA (N = 24), AC (N = 28) and CC (N = 12) using real-time quantitative PCR. Expression of *IL12B* was normalized for each individual separately using *18S rRNA* as an endogenous control. The median and distribution of *IL12B* relative expression in terms of ΔCt for *IL12B+1188* AA, AC and CC genotypes was presented in Figure 1C. No significant difference (P value = 0.165) in transcript levels of *IL12B* gene was observed between genotype groups representing AA (^median^ ΔC_t_±S.D = 17.98±1.40) and AC (^median^ ΔC_t_±S.D = 18.74±1.67). On the other hand, the median level of *IL12B* expression of AA (5 fold; P value = 0.005) and AC (9 fold; P value = 0.0001) genotypic categories were significantly lower both separately as well as collectively (7 fold, P value = 0.0001) compared to that (^median^ ΔC_t_±S.D = 15.49±1.25) of non-risk CC genotype group (Figure 1C & D).

**Figure 4:**
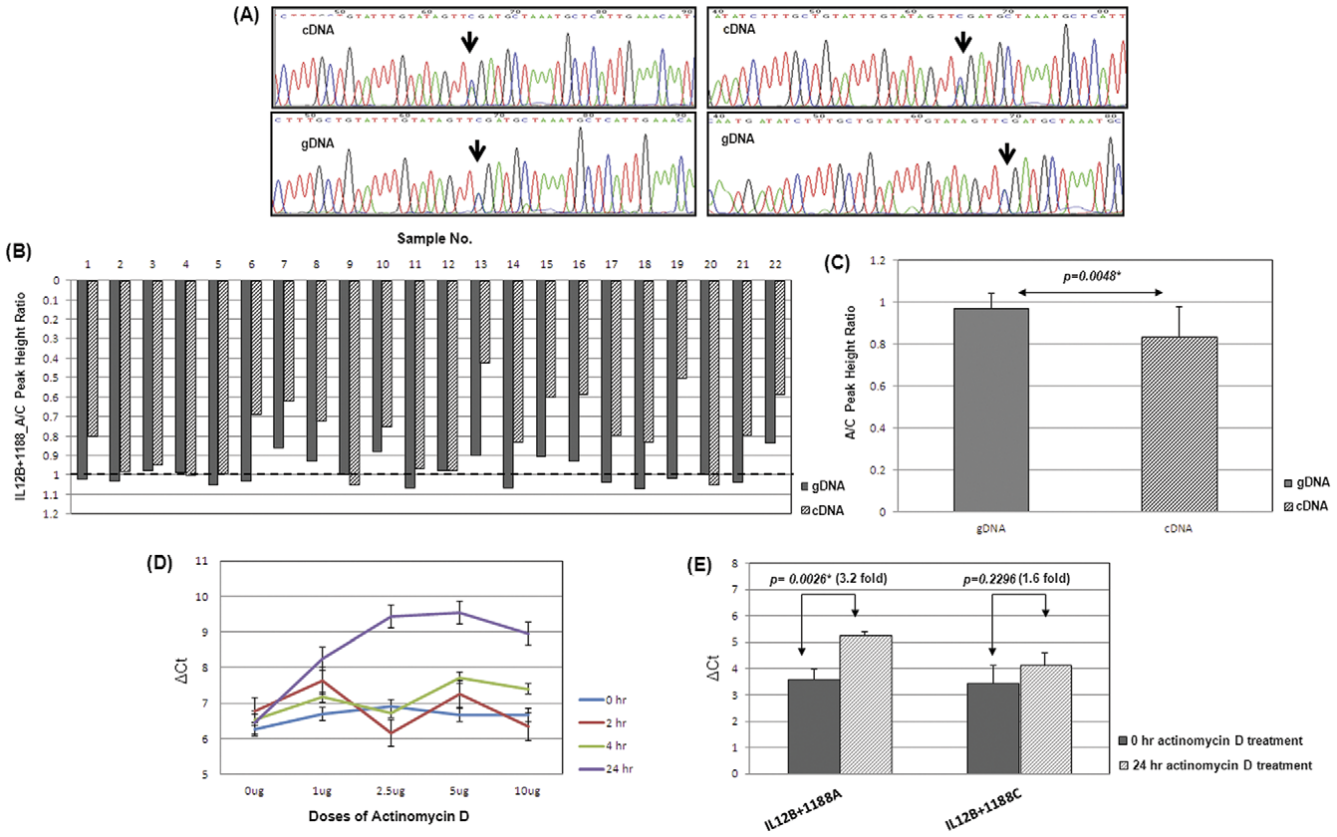
*IL12B* 3′UTR based AEI analysis. (A) Representative electropherograms showing the peak heights of the sequence encompassing *IL12B+1188* locus in gDNA (lower panel) and cDNA (upper panel) extracted from the heterozygous (AC) patients' PBMC samples. The arrowhead indicates the +1188 site. Here A and C alleles are represented as green and blue peaks respectively. Difference in peak heights in sequences between cDNA and gDNA was evident. (B) Bar graph displays the A/C peak height ratios in individual heterozygous samples (N = 22). The dotted line indicates the baseline where the A/C ratio is one. (C) The departure of A/C peak height ratios from baseline in gDNA and cDNA were shown in the form of bar diagram. Each bar represents the mean height and corresponding standard deviation. The statistical difference of this distribution was measured by Sign test. P value has been indicated. (D) Dose-response and time-course assay of actinomycin-D treatment by real time PCR. (E) HepG2 cells (1×10^5^cells/ml) were treated with the optimum dose of actinomycin-D (5 µg/ml), harvested after 0 and 24 hours after treatment and *IL12B* mRNA levels were measured and corrected for *GAPDH* mRNA for both the wild-type and variant pSiCHECK2-3′UTR constructs. Statistical significance was measured by t-test. (*) indicates the P values to be statistically significant.

To reinforce the hypothesis that *A+1188C* change affects the transcription levels of *IL12B* gene *in vivo*, we assayed if there was any quantitative difference of transcripts represented by A and C alleles in patients harboring heterozygote genotype. The test for allelic-expression-imbalance (AEI) was carried out by amplification and resequencing of 167 bp fragment that covered *IL12+1188* from cDNAs and gDNAs of twenty eight individuals with AC genotype. The peak heights representing A (green peak) and C (blue peak) alleles from individual electropherograms of paired samples were determined using PeakPicker software (Figure 4A). The average A to C peak height ratio ranged from 0.42–1.05 and 0.82–1.07 (Figure 4B) in cDNA and gDNA respectively and the comparison of these ratios was statistically significant (P value = 0.0048) estimated by nonparametric Sign test (Figure 4C). The allele specific expression of *IL12B* in the peripheral blood mononuclear cells (PBMCs) of malaria patients by 3′UTR polymorphism may be attributed to (i) differential transcript stability, (ii) influence of microRNAs and (iii) conformational change in RNA secondary structure.

### IL12B+1188 allele-based mRNA stability assay: Inhibition of transcription by actinomycin-D

**Figure 5:**
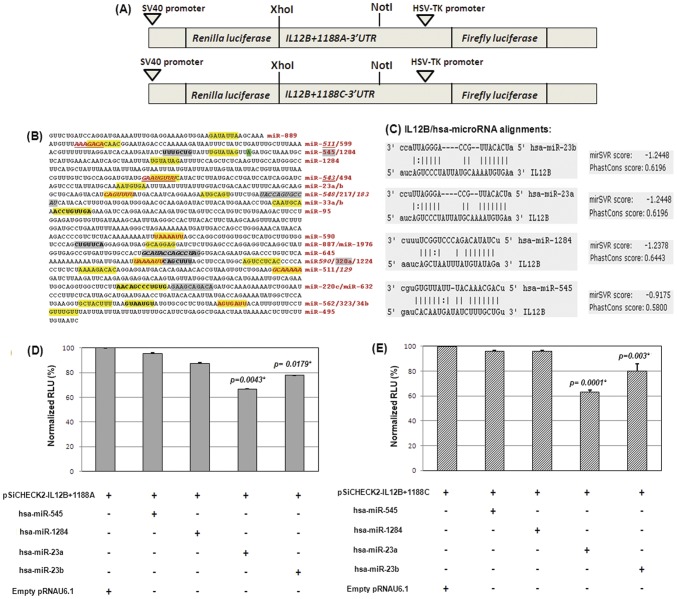
*IL12B* mRNA-microRNA interaction assay. (A) Schematic representation of reporter gene constructs for *IL12B* 3′UTR A and C alleles used for transfection assays. (B) Entire 3′UTR region was mapped for putative microRNA binding sites. The highlighted, boldfaced and underlined segments within *IL12B* sequence were the seed sequences for the miRNAs. (C) Schematic representation of the score and seed position of four miRNAs on target IL12B. (D & E) Normalized luciferase relative light units (RLU) in HepG2 cells were measured for *IL12B+1188* A and C allele containing pSiCHECK2 constructs with (+) and without (−) the effect of miRNAs. Co-transfection of the empty pRNAU6.1 (+) vector with pSiCHECK2-IL12B+1188 construct was set as 100% and reductions in luciferase expression in presence of four miRNAs were measured in relation to this. hsa-miR-23a and hsa-miR-23b resulted in significant reduction in luciferase activities for both pSiCHECK2-IL12B+1188A and pSiCHECK2-IL12B+1188C constructs. Statistical significance was measured with t-test. (*) indicates the P values and percentage reduction to be statistically significant.

In order to directly assay differences in mRNA stability between *IL12B* 3′UTR alleles, we determined mRNA levels in cells transfected with plasmids in which 1047 bp region from *IL12B* 3′UTR representing A or C (+1188) allele was cloned under Renilla luciferase gene with SV40 constitutive promoter in the vector pSiCHECK2 (Figure 5A). Twenty four hours post-transfection, HepG2 cells were treated with actinomycin-D (5 µg/ml) to suppress *de novo* transcription. Figure 4D represents the dose response and time kinetics of actinomycin-D treatment that enabled us to select for the optimum treatment conditions for the drug. mRNA levels at 0 and 24 hours after the drug treatment were quantified by real-time PCR using *IL12B* and *GAPDH* (as endogenous control) specific primers. As shown in Figure 4E, the *IL12B* mRNA levels had declined 3.2 and 1.6 folds respectively for A (ΔC_t at 0 hr_ = 3.57±0.39 vs ΔC_t at 24 hr_ = 5.23±0.17) and C (ΔC_t at 0 hr_ = 3.44±0.68 vs ΔC_t at 24 hr_ = 4.12±0.47) alleles compared to their respective starting levels suggesting that *A+1188C* polymorphism differentially influenced *IL12B* mRNA stability.

### Influence of microRNA regulators targeting IL12B 3′UTR

To identify the putative microRNA binding sites in the 3′UTR of *IL12B* gene we employed a consensus approach by using different miRNA target prediction softwares, viz. TargetScan, MiRanda, MicroCosm, miRBase and RegRNA. A comprehensive microRNA map of *IL12B* 3′UTR predicted consensusly by at least three different databases was presented in the Figure 5B. Four candidate microRNAs from this list were selected for cell based reporter gene assay as they were located in the closest proximity of the polymorphic site (hsa-miR545 and hsa-miR-1284) and conserved among species (hsa-miR23a and hsa-miR-23b). Their respective seed sequences were shown in Figure 5C.

The precursor microRNA (pre-miR) sequences for each of these four regulatory RNAs were extracted from GeneCard database followed by amplification of a region encompassing 100 bp upstream and downstream of the mature miRNA and cloning in appropriate orientation into pRNAU6.1 vector. To verify whether *A+1188C* SNP alters the binding and regulation incurred by the above candidate microRNAs, we transfected each of these pre-miR constructs in HepG2 cells along with the 1047 bp segment of *IL12B* 3′UTR containing either *+1188A* or *+1188C* variant cloned into the 3′UTR of luciferase gene in pSiCHECK2 plasmid. Reporter plasmid co-transfected with empty miR-pRNAU6.1 vector served as the control and luciferase expression was measured 48 hours post-transfection. All the experiments were done in triplicate and repeated thrice and the change of luciferase gene expression was denoted as normalized RLU (in percentage) relative to the control. Co-transfection of hsa-miR-23a (33.33%±0.08, P value = 0.0043) and hsa-miR-23b (22.22%±0.04, P value = 0.0179) with pSiCHECK2-IL12B-+1188A resulted in significant reduction in Renilla luciferase expression (Figure 5D). Similar reduction of reporter gene expression was observed when hsa-miR-23a (36.6%±1.15, P value = 0.0001) and hsa-miR-23b (20%±2.06, P value = 0.003) were transfected with pSiCHECK2-IL12B-+1188C (Figure 5E). On the other hand, luciferase activity from neither pSiCHECK2-IL12B-+1188A nor pSiCHECK2-IL12B-+1188C construct was altered by hsa-miR-545 (A = 4.35%±0.4 and C = 4.08%±0.5) and hsa-miR-1284 (A = 12.61%±0.5 and C = 4.1%±0.6). Similar trend of hsa-miR-23a and hsa-miR-23b mediated reduction of luciferase activity from pSiCHECK2-IL12B-+1188 A & C constructs were observed in HCT116 and U937 cells (data not shown). Taken together, our results suggest that hsa-miR-23a and hsa-miR-23b bind to *IL12B* 3′UTR, however, none of them exerts any allele specific influence over the *IL12B* expression.

### Allele dependent conformational changes in RNA secondary structure

**Figure 6:**
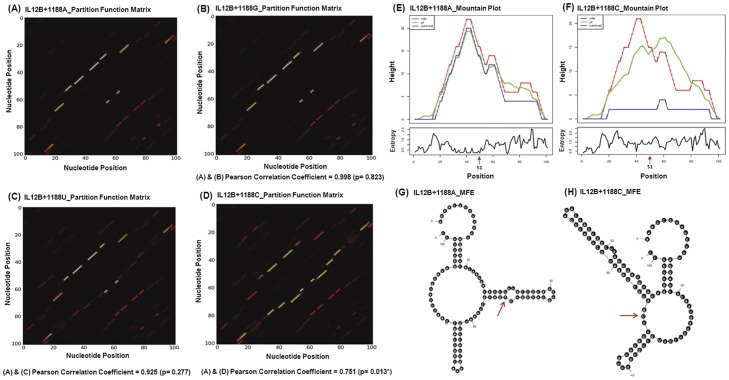
*IL12B* 3′UTR polymorphism based RNA ensemble structures. (A–D) display the SNPfold derived partition function heat maps generated for 101 nucleotide sequences harboring A (risk), G, U and C (non-risk) alleles respectively at the 51^st^ position. The partition function matrix illustrates the base-pairing probabilities represented by dots. We estimated the pairwise Pearson correlation coefficient with respect to wild-type A allele and P values to quantify the overall modulation in the RNA structural ensemble caused by a mutation. (*) indicates the P value to be statistically significant. (E & F) show the mountain plot diagrams for *IL12+1188*A and C allele for 101 bases using RNAfold. The upper panel demonstrates the height vs position graph in which the red, green and blue lines depict the minimum free energy structure, the partition function of all possible RNA secondary structures and ensemble centroid structure respectively. The lower panel represents the entropy vs position profile and arrowhead denotes the 51^st^ position, the location of *A+1188C*. (G & H) shows allele specific minimum free energy (MFE) conformations generated from RNA MFOLD. Numbers indicate the base position while the arrow directs the position of the polymorphic site.

There is increasing evidence that *cis*-regulatory RNA structural elements within mRNAs mediate post-transcriptional gene regulation by determining several aspects of the mRNA life cycle such as stability, localization, and translational efficiency [67], [68]. However, RNA structures are dynamic and evolved to adopt multiple conformations forming an ensemble that may be best described by a partition function defined as the probabilities of all possible base pairings [69]. To predict if *A+1188C* polymorphism provides any localized effect on the structural ensemble of *IL12B* mRNA, we reconstructed foldings of 101 nucleotide RNA sequences using four different bases (A, G, U and C) in the central 51^st^ position, keeping 50 nucleotides upstream and downstream sequences with respect to +1188 site identical. The partition function matrices computed for all four sequences using SNPfold were represented as dot-plots where probability of each base-pairing was denoted by a dot (Figure 6A to D). The pairwise Pearson correlation coefficients of A to G, A to U and A to C were 0.998, 0.925 and 0.751 respectively indicating a significant (P value = 0.013) modulation of overall structural assembly for A to C change (Figure 6A to D).

Mountain plots were generated for 101 nucleotide sequences harboring A and C alleles (Figure 6E & F). A mountain plot represents the secondary structure in a plot of height versus position where the height m(k) is given by the number of base pairs enclosing the base at position k. The red, green and blue curves represent minimum free energy structure, thermodynamic ensemble of RNA structures and the centroid structure respectively. The overlap between the red and green lines in Figure 6E suggested that structure produced by A allele was closer to that predicted by MFE. The blue plateau in Figure 6F indicated the central tendency of the C allele to remain in open conformation. Deviation from the base line in the position versus entropy graph of C allele shown in lower panel signified a higher randomness and positional entropy in the local structures involving C allele (Figure 6F). This also gave rise to a higher Ensemble Diversity (ED) in the structural constellation generated by C at position 51 (ED_A_ = 20.73 vs ED_C_ = 34.06). Taken together, the *in silico* modeling of RNA secondary structure indicated an overall conformational dissimilarity in the base-pairing probabilities of the RNA thermodynamic ensemble marked by A and C alleles which could result in an alternative *cis*-regulatory functions mediated by *IL12B* 3′UTR polymorphism.

## Discussion

Malaria epidemiology studies have shown that, within human populations, a high degree of variation exists between individuals with respect to malaria susceptibility phenotypes, including parasite load, disease incidence, severity, and the magnitude and type of immune responses to malaria antigens [70]. Immune response to malaria has been an object of extensive investigation aiming at understanding the genetic regulation since an inappropriate immune response leads to uncontrolled parasite replication and is detrimental to host fitness [71]. A large body of the literature has demonstrated that protection against severe *P. falciparum* malaria is provided by distinct genetic traits, while relatively less is known about the mild events which clearly constitute a substantial fraction of global socioeconomic burden of malaria [72]–[74]. To understand the role of host genetics in the susceptibility and clinical course of mild malaria, we in this study, primarily rely on reconstruction of genetic interactions among toll-like receptors (*TLR*s) and pro-inflammatory cytokine genes together with an indepth functional appraisal of determinants associated with variation in blood parasite level.

Over the past several years, genetic epidemiology studies technologically have progressed from investigating few candidate markers to interrogating thousands of variants in genome-wide association studies (GWAS) while the data analysis mostly focuses on detecting single SNP effects [75], [76]. Looking beyond the boundary of additive inheritance of SNPs using marker by marker approach may offer a better exercise to identify genetic determinants and biological mechanisms involved in disease etiology. Keeping this in mind, we have used MDR to reduce the dimensionality of the multilocus genetic data pertaining to immune response components to identify combinations of polymorphisms associated with the risk of high parasite load. MDR introduced by Ritchie, et al. is a computational strategy to detect and characterize gene-gene interactions that may be associated with disease susceptibility [41]. So far, MDR and its extensions such as GMDR, FAM-MDR, MDR-PDT, EMDR [77]–[80] have identified many interacting genetic variants underlying a wide variety of complex human diseases such as Alzheimer's disease, asthma, atrial fibrillation, autism, bladder cancer, hypertension, nicotine dependency, prostate cancer, schizophrenia, sporadic breast cancer and type II diabetes [81]–[89]. In our study single SNP and multifactor dimensionality reduction analyses together identified three genetic loci which served as important factors in controlling blood parasitemia in mild malaria. These loci include *TLR4Thr399Ile*, *TNF-1031* and *IL12B+1188*. Two additional loci viz. *LTA80* and *TLR9P545P* appear in combination with *TLR4Thr399Ile* and *IL12B+1188* suggesting that the formers fail to show statistically significant association in independent analyses because of their minor effects on trait variation (Table 3) [90]. Notably, the minor allele frequencies of both *LTA80* and *TLR9P545P* were approximately 40% in our samples [21]. The presence of *IL12B+1188* in five of six models and elevation of odds ratio of *TLR4Thr399Ile*, *TNF-1031* and *LTA80* in combination with *IL12B+1188* are suggestive of a pivotal genetic influence of *IL12B* on malaria phenotype. This is supported by the fact that different malaria phenotypes such as hyperparasitemia, severe malarial anemia or cerebral malaria are often found to be associated with *IL12B* gene [34], [91]–[94] while the genetic alliance between parasitemia with *LTA* is seldom unequivocal [95]–[97]. A study conducted among 198 individuals belonging to 34 families living in Burkina Faso using the pedigree-based generalized multifactor dimensionality reduction approach, identified statistical interactions among immune genes including *IL12B* 3′ untranslated region, *IL12Bpro*, *LTA+80* for mild malaria, maximum parasitemia or asymptomatic parasitemia [98] underscoring the usefulness of modeling gene-gene interaction in genetic dissection of complex diseases (Table 3).

The association of high parasitemia with *IL12B+1188* (Table 1) and significantly low expression of the cytokine in individuals harboring risk genotypes (Figure 1) indicate that the susceptible individuals are deficient in controlling parasite replication due to inadequate level of *IL12B* transcript in mild malaria patients. Critical role of *IL12B* in malaria has been demonstrated by other studies that show *IL12* production is inversely associated with disease severity in human malaria [94], [99], [100] and the molecule is extremely effective in correcting malarial anemia in murine model [101], [102]. Reduced expression of *IL12B* and *LTA* under *TLR4* risk genotype background may be attributed to altered *TLR4* signaling (Figure 2). To the best of our knowledge, this is the first report that provides functional validation of statistical epistatic models demonstrating *in vivo* suppression of cytokine gene expression due to genetic deficiency of *TLR4* signaling. The attenuated *TLR4* signaling by the variant receptors may be ascribed to altered distribution of electrostatic surface potential as shown by homology modeling in our previous study [21]. However, it should be borne in mind, a comprehensive profiling of transcriptome and network analyses are necessary to identify additional genetic partners involved in the pathway.

Pinpointing phenotypically causal variants from a large fraction of SNPs that show statistical association to diseases remains a major challenge. Only a handful of disease-associated variants occur at non-synonymous (nsSNPs) sites while a majority are located on the non-coding genomic regions (ncSNPs) adjacent to protein coding genes suggesting that the latter may be involved in transcriptional regulation. Global expression quantitative trait loci (eQTL) analyses in yeast, mice, and humans have detected significant levels of *cis* and *trans*-eQTL that simultaneously regulate a large fraction of the transcriptome [103]–[108]. We previously reported that the polymorphism located at *TNF-1031* displayed significant association with peripheral parasite load [21]. Here we showed that any variation from the *TNF-α* wild-type haplotype resulted in reduced promoter efficiency and the maximal reduction was observed for the haplotype that was altered at −1031 locus. The dysfunctioning of *TNF-α* promoter due to −1031 polymorphism may be attributed to allelic differences in binding of transcription factors (TFs). A number of transcription factor binding sites (TFBSs) were abolished in presence of the variant allele (C) which assumed a sterically inaccessible locked-in conformation as depicted in the bioinformatic outputs (Figure 3D–F). Chromatin immunoprecipitation or electrophoretic mobility shift assays (EMSA) are necessary to validate *in-silico* prediction of altered binding affinity between putative TFs and promoter SNP. Reduction of transcriptional activity due to single nucleotide variation in *TNF-α* promoter has been reported for the polymorphisms located at −863 [109], −376 [110] and −238 [111].

Allelic-expression-imbalance (AEI) phenomenon which serves as an integrative quantitative measure of any and all *cis*-acting regulatory variants [112] is highly context-specific [113] and widespread not only in humans and mice but in most organisms [114]–[120]. It can affect the transcriptome by altering stability [121], processing efficiency [122], isoform expression of mRNAs [123], [124] and inducing epigenetic changes [125], [126]. The unequal expression of *IL12B* between marker genotypes and allelic imbalance detected in malaria patients' PBMC samples strongly suggest that *IL12B+1188* polymorphism executes a *cis*-regulatory function. To interpret the probable mechanisms of *IL12B+1188* mediated AEI, we have investigated allele specific transcript stability, differential interaction of *IL12B* 3′UTR with candidate microRNAs and allele-dependent modulation of RNA sub-optimal structures. The remarkable modulation of local RNA secondary structures due to A to C transversion observed in this study may affect the transcript stability directly or through the association of RNA-binding proteins (RBPs) indirectly [69]. Many RNA binding proteins have both sequence-specificity and RNA-secondary structure binding preferences and are known to co-regulate functionally related transcripts [127]. From SNP-targeted studies, it has been estimated that 20% of the measured transcripts show 1.5 fold differences between alleles while 30% show 1.2 fold differences [118], [128]. However, it is still obscure how small allelic imbalance may cause a phenotype and how accurate the bioinformatic predictions are with actual experimental evidences.

Recent studies have also shown that 3′UTRs contain recognition motifs for microRNAs (miRNAs) which play important gene-regulatory roles by pairing to their target mRNAs to direct posttranscriptional repression. The most accurate predictors of miRNA target sites relies on conserved matches to the ∼7 bp seed region near the 5′end of the microRNA and also on the accessibility of target sites. The four candidate microRNAs (hsa-miR-545, hsa-miR-1284, hsa-miR-23a and hsa-miR-23b) predicted in common from multiple databases target *IL12B* 3′UTR sequence located proximally to *A+1188C*. Our data showed hsa-miR23a and hsa-miR-23b interacts with *IL12B* 3′UTR to repress gene expression without any allelic bias. This may be attributed to our reductionist approach to candidate microRNA prediction and limited ability of the current prediction algorithms that calculate target efficacy based on interactions between the mRNA with itself and the mRNA with a miRNA. This is of particular importance because mRNA-miRNA interaction occurs in a complex cellular environment in which mRNAs and miRNAs are likely to be bound by cellular RNA-binding proteins, which are currently impossible to account for *in silico*. Our findings exemplify that diversity of posttranscriptional gene regulation may extend beyond microRNAs and underscores the importance of characterization of structural *cis*-regulatory elements and their interaction partners in the context of mRNA stability. Recently, there has been renewed interest in identifying the effects of disease-associated noncoding SNPs on changes in RNA structure [129], [130] which may regulate gene expression at virtually every possible stage ranging from local chromatin remodeling to mRNA translation [131]–[133]. Taken together, our findings convincingly demonstrated inadequate gene expression of pro-inflammatory cytokines, namely *IL12B* and *TNF-α* attributable to *cis*-regulatory polymorphisms together with loss of efficacious *TLR4* mediated signaling due to nsSNP contribute to uncontrolled parasite growth in *P.falciparum* malaria in an epistatic manner. In the study of genetics of complex diseases, to determine which of the multitude of variants carried by an individual are responsible for a given phenotype remains a massive task. Our study emphasizes the need to evaluate gene-gene interaction and biological credibility of hundreds of common non-coding variants with low effect size as powerful complementary research strategies to illuminate the genetic architecture of common diseases.

## Supporting Information

Table S1 **Oligonucleotide sequences.**
(DOC)
