# Supplementary material for: Gene-Gene Interaction and Functional Impact of Polymorphisms on Innate Immune Genes in Controlling Plasmodium falciparum Blood Infection Level
Source: PLoS One. 2012 Oct 12;7(10):e46441. doi: 10.1371/journal.pone.0046441 (PMC3470565; doi:10.1371/journal.pone.0046441)
Supplement: Table S1 — Oligonucleotide sequences. (DOC) [file pone.0046441.s001.doc]

**Supporting Information**

**Table S1: Oligonucleotide sequences**

| **Sl No.** | **Primer ID** | **Oligonucleotides (5’–3’) sequence** |
| --- | --- | --- |
| 1 | *IL12Bpro F.P* | ATGTACAGCCTGTCTCCGAGAGAA |
|  | *IL12Bpro R.P* | TCTCTGCCCCTCGGGACTGACTAT |
| 2 | *IL12B+1188 F.P* | CTTCCAGGTTCTGATCCAGGATGA |
|  | *IL12B+1188 R.P* | CTGATGTACTTGCAGCCTTGCTTGA |
| 3 | *IL12Bexp F.P* | AGACACAACGGAATAGACCC |
|  | *IL12Bexp R.P* | ATGGCAACTTGAGAGCTGGA |
| 4 | *LTAexp F.P* | AGATGCATCTTGCCCACAGCA |
|  | *LTAexp R.P* | GTAGACGAAGTAGATGCCACT |
| 5 | *18S rRNAexp F.P* | GTAACCCGTTGAACCCCATT |
|  | *18S rRNAexp R.P* | CCATCCAATCGGTAGTAGCG |
| 6 | *GAPDHexp F.P* | GACATCAAGAGGGTGGTGAAGCAG |
|  | *GAPDHexp R.P* | CACCCTGTTGCTGTAGCCATATTC |
| 7 | *TNFα_MluI_F.P* | TGTACGCGTGTCTGGGAGTGAGAACTTAA |
|  | *TNFα_XhoI_R.P* | AGTCTCGAGCCCTCTTAGCTGGTCCTCAG |
| 8 | *IL12B_XhoI_F.P* | ACCCTCGAGGCTACTATAGCTCATCTTG |
|  | *IL12B_NotI_R.P* | TTGGCGGCCGCCTTGAAAAGTTGTCAGTA |
| 9 | *hsa-miR545_BamHI_F.P* | ACAGGATCCTCGGCCATTATAATACAACCT |
|  | *hsa-miR-545_HindIII_R.P* | CAGAAGCTTTTATCACTCAAGTCCCACCA |
| 10 | *hsa-miR-1284_BamHI_F.P* | ACCGGATCCAATGAAAATATTCCTGCAG |
|  | *hsa-miR-1284_HindIII_R.P* | TATAAGCTTTCAGACGGTCTTGCCTAGT |
| 11 | *hsa-miR-23a_ BamHI _F.P* | TCTGGATCCCATTACCTCCTTTGCTCTCT |
|  | *hsa-miR-23a_ HindIII _R.P* | ACTAAGCTTGTGAACACGACTTGGTGTGG |
| 12 | *hsa-miR-23b_ BamHI _F.P* | AGGGGATCCATGCCAACTAAACGAACAAC |
|  | *hsa-miR-23b_ HindIII _R.P* | TAGAAGCTTAGAGGTCATCGCTGGGCATA |
